# The Storm Within: Exploring First-Year Nursing Students’ Experiences of the Objective Structured Clinical Examination

**DOI:** 10.1177/23333936251400692

**Published:** 2025-12-06

**Authors:** Marko Stojiljkovic, Line Nortvedt, Kari Röhrl, Elisabeth Hessevaagbakke, Katrin Lindeflaten, Anne-Marthe Rustad Indregard, Daniela Lillekroken

**Affiliations:** 1 Oslo Metropolitan University, Norway

**Keywords:** assessment, focus groups, nursing students, objective structured clinical examination, qualitative analysis, Norway

## Abstract

Although the Objective Structured Clinical Examination (OSCE) is widely used in nursing education as a summative assessment of students’ learning and clinical competence, less attention has been given to students’ experience of the examination process. The aim of this study is to explore first-year nursing students’ experiences with the OSCE. Data were collected through eight focus group interviews with first-year students between March and June 2025 and analyzed using qualitative content analysis. The analysis resulted in three main categories: “Before the OSCE – The Calm before the Storm,” “During the OSCE – In the Eye of the Storm,” and “After the OSCE – Emerging from the Storm.” Students described the examination as both empowering and stressful. For many, it validated growth, enhanced confidence, and motivated further learning. For others, it raised concerns about fairness and caused frustration. The OSCE was experienced as a “storm” of emotions that strengthened skills, resilience, and students’ professional identity, while also causing stress and raising feelings of inequity. Furthermore, the study highlights the need for improved student preparation, educators’ feedback, and alignment between academia and clinical practice.

## Introduction

Today’s learning landscape offers more options than ever, but it also presents more challenges. The new generations of young people adopt new learning styles during their education; therefore, university educators need to adapt not only to socio-cultural changes but also to generational shifts in teaching and assessment methods (Rosa-Castillo et al., 2022). In nursing education, assessment is essential for monitoring the quality of educational programs but is also used to gather information about student learning, evaluate competencies, and assess clinical performance (Oermann & Gaberson, 2019). According to Mislevy (2018), assessment involves collecting information about what students know and can do, as well as providing feedback to students. This feedback is important in clinical practice as students develop their competencies and learn to navigate complex clinical situations. For an effective assessment, Brookhart and Nitko (2019) suggest five guidelines to consider when choosing and implementing assessment strategies in the classroom, online courses, skills or simulation learning environments, or clinical settings: (i) identify the learning objectives (outcomes or competencies) to be assessed, (ii) match the assessment strategy to the learning goal, (iii) meet the students’ needs, (iv) use multiple assessment strategies and indicators of performance for each outcome, and (v) remember the limitations of assessment when interpreting the results.

To the best of our knowledge, there are no specific requirements for assessment methods for nursing students completing courses. However, there are several assessment strategies that nurse educators can use to collect information about students’ learning and performance, regardless of where learning takes place. These methods include tests that can be developed using various types of items, essays, other written assessments, projects, small-group activities, oral presentations, e-portfolios, performance observations, simulation-based assessments, objective structured clinical examinations (OSCEs), and conferences, among others (Oermann & Gaberson, 2019).

Tests can be administered at the start of a course or instructional unit to assess whether students possess the prerequisite knowledge necessary for achieving the learning outcomes. The results can reveal gaps in learning and performance that should be addressed first (Oermann & Gaberson, 2019). Measurement involves assigning numbers to represent student achievement or performance. These scores indicate the extent to which a student exhibits a particular characteristic. However, one challenge is that not all outcomes can be measured through testing; therefore, many outcomes are assessed qualitatively using other methods, such as observations of student performance in clinical practice or simulation (Oermann & Gaberson, 2019). One such method is the Objective Structured Clinical Examination (OSCE) (Oermann & Gaberson, 2019). The OSCE is crucial in education to determine readiness and competence for certification and accreditation (Arrogante et al., 2021). Accordingly, the OSCE is frequently used as a summative evaluation to assess students’ clinical competence (Harden et al., 1975). As a result, the OSCE has been adopted by educational institutions as a valid and reliable assessment method (Arrogante et al., 2021).

In clinical nursing education, simulation-based education provides an excellent opportunity to not only teach competencies through observed competency-based methods but also assess these competencies. Simulated-based assessment is designed to evaluate various professional skills, including knowledge, technical and clinical skills, communication, and decision-making, as well as higher-order competencies such as patient safety and teamwork skills. The main simulation-based assessment strategies are both formative and summative evaluations. According to Brookhart and Nitko (2019), formative evaluation assesses students’ progress in meeting the desired outcomes and developing clinical competencies. In this sense, formative evaluation includes an ideal phase to achieve the strategy’s purpose: debriefing.

The International Nursing Association for Clinical Simulation and Learning (2016) defines debriefing as a reflective process immediately following the simulation-based experience where “participants explore their emotions and question, reflect, and provide feedback to one another.” In contrast, summative evaluation is used to establish the learning outcomes achieved by students at the end of the course. This evaluation strategy is helpful to educators in evaluating students’ decisions, skills, communication, teamwork, and other competencies (Oermann & Gaberson, 2019). Additionally, the summative evaluation is crucial for determining readiness and competence for certification and accreditation (Brookhart & Nitko, 2019). Accordingly, the Objective Structured Clinical Examination (OSCE) is commonly conducted in simulation-based assessment as a summative evaluation to evaluate students’ clinical competence (Harden et al., 1975). Consequently, the OSCE has been used by educational institutions as a valid and reliable assessment method (Arrogante et al., 2021).

In the Bachelor of Nursing program at Oslo Metropolitan University (OsloMet, 2024a), both formative and summative assessments are essential components of most courses. These assessments are used across various learning environments, including classrooms, clinical settings, and simulation-based contexts, and are intended to work in combination. Formative assessment plays a particularly significant role, as it provides students with feedback that helps them enhance their learning.

The most commonly used learning model for nursing students during their clinical placements is what Lave and Wenger (1991) refer to as the “apprenticeship model.” According to Lave and Wenger (1991), learning involves not only acquiring new skills, tasks, and functions but also developing new understandings, all of which are embedded within a social context, such as a nursing home. In this setting, students receive daily supervision and formative evaluation from a nurse preceptor. Additionally, they participate in weekly seminars led by a university-based nursing educator, where they reflect on and discuss the skills, tasks, and functions they have acquired as part of the formative assessment process. Students are also required to complete an individual written assignment and conduct self-evaluations at both the midpoint and the end of their clinical period. Ongoing feedback from the nurse educator supports the students in developing the knowledge and competencies necessary for future professional practice. However, challenges remain in providing effective feedback related to clinical performance when students are assessed through summative evaluation. To ensure accurate and meaningful assessments, further understanding and development of evaluation methods are needed.

## Implementing the OSCE – Assessing Students’ Knowledge, Skills, and Overall Competence

In spring 2023, students from the 2022 cohort enrolled in the “Supplementary Education for Nurses Educated Outside the EU/EEC” (SKOMP course) at OsloMet became the first group to undergo summative assessment using the OSCE (OsloMet, 2022). These students provided feedback on their experience with the new assessment format. Their responses indicated that they had positive experiences with this type of summative evaluation.

From March 2025, the Department of Nursing and Health Promotion at OsloMet adopted the OSCE as an assessment method after first-year students completed the course Foundations of Nursing 2 (OsloMet, 2024b), a 7-week clinical placement in nursing homes, hence replacing the 4-hour written school exam as a summative assessment. The department’s intention in replacing the 4-hour written school exam was to ensure higher quality in education by strengthening the connection between theoretical knowledge and practical training from clinical placements, as well as enhancing the overall learning experience. To achieve this, the course coordinators focused on aligning the course content more closely with practice and the competencies students develop at the department’s simulation learning environment. Additionally, we sought to increase the variety of assessment methods within the program curriculum to better support students’ diverse learning needs and provide a more comprehensive evaluation of their skills.

The development of the OSCE at OsloMet was inspired by international models of competence assessment in nursing education (Goh et al., 2022; van der Vleuten & Schuwirth, 2005). The process followed a structured, iterative approach focusing on validity, reliability, feasibility, and educational impact. The initial phase involved constructing an OSCE blueprint that aligned each station with the bachelor’s program’s learning outcomes and national nursing competence standards. The aim was to ensure a broad sampling of clinical competence, including communication, patient safety, clinical reasoning, and technical skills. The final blueprint consisted of multiple stations that represented authentic clinical scenarios, reflecting clinical course learning outcomes. Each student rotated through three stations, with a designated time for scenario review and performance. To enhance objectivity and minimize rater bias, all nurse educators employed at the department who served as evaluators participated in calibration workshops prior to implementation. These sessions included review of performance criteria, scoring practice, and discussions of expected competence levels. This process ensured shared understanding and inter-rater consistency across stations, similar to the psychometric calibration described by Goh et al. (2022).

A pilot OSCE was conducted to test logistics, timing, and clarity of instructions. Feedback from both students and examiners was systematically collected and analyzed, and an in-depth debrief was conducted. Based on these results, station instructions, checklists, and timing were refined to improve authenticity and flow. This multi-phase development process ensured that the OSCE at OsloMet was pedagogically grounded, psychometrically sound, and aligned with van der Vleuten and Schuwirth’s (2005) utility framework, striking a balance between validity, reliability, acceptability, and cost-effectiveness.

The OSCE consisted of two components: a theoretical multiple-choice test with 40 questions, requiring a minimum of 24 correct responses (60%) to pass, and a practical examination comprising three simulation stations where students performed clinical nursing procedures. These stations allowed students to demonstrate their ability to carry out essential clinical tasks. Together, the two components enabled assessment of both theoretical knowledge and procedural competence in a realistic and structured way.

For the first group of students (the A and B classes), the three stations included: (i) measuring vital signs. Students performed a full assessment of pulse and blood pressure on a patient actor. They were evaluated on communication, patient identification, obtaining consent, correct measurement technique, and interpretation of findings, (ii) subcutaneous injection in the abdomen. Here, students were assessed on whether they verified the medication, maintained aseptic technique, selected the correct injection site, communicated effectively with the patient, and safely administered the injection, and (iii) use of sterile gloves, a sterile procedural task, where correct use of gloves, hand hygiene and ergonomic techniques were also evaluated. Later, for the second group (the C and D classes), the use of sterile gloves was replaced with cardiopulmonary resuscitation (CPR), with a defibrillator performed on mannequins. Here, the students’ hands-on skills, such as chest compression quality, timing of ventilations, and correct use of a defibrillator, were evaluated consistently using objective criteria.

At each station, students were assessed not only on the technical execution of the procedure but also on communication, introduction to the “patient,” obtaining consent, proper hand hygiene, use of correct equipment, and adherence to sterile or hygienic principles. Each scenario was time-limited to 8 min, simulating real clinical pressure. This required students to integrate theoretical knowledge with practical skills efficiently and accurately.

To successfully pass the practical part of the OSCE, students were required to achieve a minimum of 60 % correct responses on the scoring sheet for each individual station. Any action that posed a risk to patient safety or to the student’s own safety resulted in an automatic failure. Such critical errors, so-called “red flags,” included contaminating a sterile needle or sterile gloves, performing an injection without verifying the medication or patient identity, or breaching aseptic technique in a manner that could lead to infection. In these cases, the student failed, and it was required to repeat the entire examination cycle. This strict safety criterion reflected the program’s emphasis on cultivating professional responsibility, situational awareness, and patient-centered care as fundamental components of nursing competence. As the OSCE functioned as a high-stakes summative assessment of students’ knowledge and skills, post-exam debriefing was not routinely incorporated at the end of the OSCE.

From an academic perspective, there is limited research on nursing students’ experiences with the OSCE as a summative assessment method for evaluating knowledge, skills, and overall competence in clinical courses such as Foundations of Nursing 2. Notably, OsloMet is the first institution in Norway to implement the OSCE in the first year of bachelor’s level nursing education. This study aims to fill that gap by offering insights that can inform the development of the OSCE scenarios and checklists in nursing programs. In doing so, it also aims to enhance students’ perceptions of summative assessment, ultimately supporting their readiness to meet the challenges of contemporary healthcare and deliver high-quality, patient-centered care across diverse clinical settings.

## Methods

### Aim of the Study

The aim of the study was to explore nursing students’ experiences with OSCE as an assessment method of their skills and competencies after completing Foundations of Nursing 2. The research question that will be answered is as follows: What are nursing students’ experiences with OSCE as a summative assessment method for evaluating their knowledge, skills, and general competence after completing the Foundations of Nursing 2 course?

### Design

This study employed an Exploratory–Descriptive Qualitative design, which is grounded in a constructivist/interpretivist epistemology that assumes multiple realities are constructed through individuals’ experiences (Hunter et al., 2019). This type of design is useful when little is known about a phenomenon, and the goal is to explore and describe participants’ perspectives to generate contextually based understanding rather than to test existing theory. Because the use of OSCEs as a summative assessment for first-year students is not well documented, an exploratory-descriptive qualitative approach allowed us to examine how students interpreted and made meaning of their evaluation experience. This design therefore aligns directly with our research aim of understanding students’ perceptions to inform and improve assessment practices.

### Recruitment

There are no specific criteria or rules for determining the sample size in qualitative research (Berg & Lune, 2012). However, it is essential to involve participants who are familiar with and knowledgeable about the research topic; therefore, to obtain rich information about the topic of interest, a purposeful sample was chosen (Berg & Lune, 2012). All students enrolled in the bachelor’s program in nursing at OsloMet (Pilestredet campus) (*N* = 550) were invited to participate. The program is divided into four classes (A, B, C, and D), with AB being the first to undergo the OSCE assessment. Recruitment was initiated by course coordinators, who informed all four classes about the study both verbally and by email. This was followed by several reminder emails sent by nurse educators and researchers involved in the project. The study was conducted in the simulation learning environment at the Faculty of Health Sciences, Department of Nursing and Health Promotion, OsloMet.

### Sample Characteristics

Participants were eligible for inclusion in the study if they were first-year students enrolled in the bachelor’s program in nursing at OsloMet and had successfully completed the Foundations of Nursing 2 course at a nursing home. The participants in the first phase of data collection were students from AB classes. Out of 257 students in AB classes, only 19 expressed interest and agreed to participate. The participants, consisting of nine women and ten men, ranged in age from 19 to 45 years, with a mean age of 24.6 years. They took part in four focus group interviews organized as follows: one group had six participants, another five, and the remaining two groups had four participants each.

The participants in the second phase were students from CD classes. Out of 228 students, only 22 participated in the second phase of data collection. Eight men and fourteen women, aged between 19 and 45 years, with a mean age of 24.8 years, participated in four focus groups. The number of participants in each focus group was as follows: one group had three participants, another had four, a third had five, and the last had ten.

Out of all 41 participants, 16 had no previous work experience in healthcare. The remaining participants had, on average, between 6 months and up to 10 years of work experience across various healthcare settings. To ensure diverse perspectives, both students who passed and those who failed the OSCE were included.

### Data Collection

Data were collected through eight focus group interviews with 41 first-year students as participants. Focus groups were chosen because collective discussion enables participants to build on one another’s comments, negotiate meanings, and articulate shared expectations and norms (Barbour, 2018). Since OSCEs are conducted within a shared educational and social setting, the interactive nature of focus groups enabled deeper insight into how students collectively interpreted and understood the summative assessment of their skills and knowledge after completing the Foundations of Nursing 2 course. Employing focus group interviews as a data-collection method provided a richer understanding than would have been possible through individual interviews alone.

The data were collected in two phases: Phase 1, which took place in March 2025, involved students from AB classes, while Phase 2, which took place in June 2025, involved students from CD classes. During Phase 1, four focus group discussions were held over a 3-week period, spanning from March to April 2025. In Phase 2, four focus group interviews were conducted over a 2-week period in June 2025.

Depending on the depth of discussions within each group, the focus group interviews lasted between 31 and 60 min. The focus groups were held in a quiet classroom at the university after the students had completed the summative evaluation. All focus group interviews were audio-recorded using the Diktafon app, developed by the University of Oslo, Norway, to ensure secure audio recordings. The app integrates with the Nettskjema service, allowing researchers to securely upload, manage, and transcribe audio files verbatim.

Each focus group was facilitated by a moderator and a co-moderator, all of whom were employed as nurse educators at OsloMet’s Department of Nursing and Health Promotion. The research team consists of one man and six women, with extensive experience in nursing education and qualitative research (as Associate Professors and Lecturers). All the researchers contributed to data collection.

### Interview Guide

The focus group interviews were guided by an interview guide developed through a review of relevant literature on OSCE. The development of the interview guide consisted of five phases: (i) identifying the prerequisites for using semi-structured interviews; (ii) retrieving and using previous knowledge; (iii) formulating the preliminary semi-structured interview guide; (iv) pilot testing the interview guide; and (v) presenting the complete semi-structured interview guide (Kallio et al., 2016). The complete interview guide and questions used in the focus groups are presented in Table 1.

**Table 1.** Interview Guide.

*Introduction* • Welcome and introduction of the moderator/co-moderator. • Information regarding the purpose of the interview. • Information regarding anonymity, voluntary participation, and the option to withdraw without negative consequences for their further education at the university. • Brief round of participant introductions (age, gender, and work experience). *Theme 1: Preparation for the OSCE examination* 1. Could you describe how you prepared for the OSCE? 2. How did you find the information you received prior to the OSCE? 3. What experiences have you had with various learning resources, such as curriculum material, supervision within the department’s simulation learning environment (SLE), use of VAR (electronic procedure manual), or digital platforms like YouTube? Follow-up: • Which of these did you find most helpful? 4. Is there anything you wish you had handled differently while preparing for the OSCE? 5. Could your teachers have done anything differently to make you feel more prepared? *Theme 2: Experiences with skills training* 1. Could you describe your experience with the skills training in the SLE before the OSCE? Follow-up questions: • Did you feel you had sufficient time to practice the various procedures? • Did you get enough support and feedback from teachers? • How did you find the clinical skills training during your nursing home placement (before the OSCE)? 2. Could you provide examples of what could have been done differently in the SLE to enhance your learning outcome before the OSCE? Follow-up: • Could you please give examples of what could have been done differently in clinical practice to enhance your learning outcome before the OSCE? *Theme 3: Experiences during the examination itself* 1. Could you describe how you experienced the structure of the exam day, such as timing, examiners, and exam tasks like multiple-choice questions? 2. Was there anything particular that made you feel more or less anxious? 3. How did you find the distribution of time and tasks at the various stations? *Theme 4: Assessment and feedback* 1. Could you describe how you perceived the assessment criteria and the fairness of the evaluation in the OSCE? 2. Could you describe your feelings about the feedback you received after the OSCE? 3. Could you provide some examples of how the feedback might have been given differently? *Theme 5: Reflection and suggestions for improvement* 1. Could you describe what you learned from completing the OSCE? 2. If you could change one aspect of the OSCE, what would it be? 3. What advice would you provide to future students preparing for the OSCE? *Conclusion* • To summarize, we have discussed . . . [brief recap]. Is there anything you would like to add about your experiences with the OSCE that we have not yet covered? Thank you very much for participating in this focus group and for sharing your experiences with us.

In addition to the prepared questions, several follow-up prompts were used to encourage participants to elaborate on their experiences or clarify potential misunderstandings. This ensured a deeper exploration of students’ perspectives. It also ensured that all participants had the opportunity to share their views and contribute meaningfully to the discussion.

### Data Analysis

The empirical data comprise 118 A4 pages of transcripts. All data were manually analyzed to gain a deeper understanding of the findings. While the primary responsibility for the analysis was assigned to the first and last authors, all co-authors actively participated by reading and interpreting the interview transcripts and providing constructive feedback throughout the process. The analysis followed a qualitative inductive content approach, based on the framework outlined by Kyngäs (2020), which involves three stages: preparation, organization, and presentation of results.

During the preparation step, the researchers listened to the audio recordings and thoroughly read the transcripts line by line, identifying and extracting sentences relevant to the study’s aim. The related sentences were classified and labelled as open code, a step that facilitated the initial reduction and structuring of the data. Any disagreements were resolved through discussions until a shared understanding was reached. During this step, the researchers returned to the raw data and confirmed that the issues identified were discussed in the context of the study’s aim and that they answered the research question. Once this has been confirmed, the researchers gathered the open codes and labelled a subcategory with an appropriate name, for example, “Engaging in preparation and developing study strategies.” Finally, in the reporting phase, the abstraction process continued until the subcategories were organized into categories based on similarities in content and among themselves. The findings were presented through detailed descriptions of each category, illustrated with direct quotes from participants. A coding tree example is provided in Table 2.

**Table 2.** An Example of the Coding Tree.

Meaning units	Sub-categories	Categories
I completed most of my preparations during my clinical placement. So, I went through most of the procedures there . . . got some responsibility to go over things, especially doing NEWS measurements . . . (P, FG4, CD classes)	Engaging in preparation and developing study strategies	Before the OSCE – The calm before the storm
The only thing we noticed was during the last week, when suddenly some teachers said different things, and then people became very uncertain and thought we were supposed to do it [a procedure] (P, FG1, AB classes)	Experiencing uncertainty and apprehension about the upcoming assessment	
It was quite a stressful start to the day. And once we were in there, your hands started to tremble a bit. You don’t really know . . . (P, FG2, AB classes)	Questioning their level of readiness and self-confidence	
But now I was really prepared . . . Last time, I was so stressed that it got the better of me. This time, I told myself I had to calm down. That’s what ruined it for me last time. So now I was very focused on not stressing myself out . . . (P, FG1, CD classes)	Managing stress and maintaining composure in high-pressure situations	
Overwhelming. It was very overwhelming because there was an examiner standing there, and the equipment was laid out, and I was like, okay, what am I actually supposed to do now? I almost had a blackout, but then it went fine. (P, FG4, AB classes)	Processing information and making decisions in real time	During the OSCE – In the eye of the storm
Many people [students] made a lot of mistakes, but still passed . . . (P, FG3, AB classes)	Evaluating the fairness and authenticity of the assessment	
I don’t think eight minutes was too little time, and two minutes for reading was fine in a way, but I don’t really understand why we should have time pressure, because in reality . . . if you spend nine minutes inserting a catheter, that’s fine. So, I don’t understand why it has to be eight minutes, because no one can manage it anyway, especially with tasks like drawing up medication. It just becomes an unnecessary stress factor with the whistle and “run here, run there, be quick there, read this . . ..” People often become completely overwhelmed and lose focus on what they should be focusing on (P, FG3, CD classes)	Reflecting on the challenges associated with the time required to perform procedures	
It’s really just about not stressing. Then you manage . . . You gain experience by seeing how it’s actually done. Everything before today was just a question mark. (P, FG2, CD classes)	Reflecting on performance and identifying key learning points	After the OSCE – Emerging from the storm
We only got an explanation (for not passing the OSCE). So [name of supervisor] couldn’t answer because they weren’t the examiner that day. Otherwise, I did get some explanation . . . (P, FG3, AB classes)	Learning from feedback to support ongoing learning, despite frustrations	
The sense of accomplishment afterwards [after the procedure] . . . It was amazing to come out of the injection station and think, “Wow, I actually did it!” . . . (P, FG1, AB classes)	Experiencing relief upon completion	

After conducting eight focus group interviews, data adequacy was assessed through ongoing evaluation of the diversity and depth of participants’ statements. While a complete range of possible experiences cannot be assumed, the sample offered rich and varied perspectives from first-year students. This ensured that a reasonable range of their experiences with the OSCE was captured to address the research question. Therefore, the researchers concluded that the data were sufficient to address the research question within the aim of this study.

### Ethical Considerations

The study is conducted in accordance with the principles outlined in the Declaration of Helsinki of the World Medical Association (2025), including obtaining informed consent, discussing potential consequences, and maintaining confidentiality. Approval to conduct the study was sought and obtained from the Norwegian Agency for Shared Services in Education and Research (SIKT/Ref. No. 176381), as well as from the leader of the Department of Nursing and Health Promotion. Following approval from both SIKT and the department’s head, the researchers provided verbal and written information about the study to all potential participants (*N* = 550). Prior to data collection, written informed consent was obtained from each participant.

All students participating in the study were informed that their participation was voluntary and that they could withdraw at any time without facing any negative consequences for their future education at the university. However, the research team members were involved in various aspects of the OSCE, which could potentially influence recruitment and student participation in the study. The last author served as the principal investigator, and the first author is the main coordinator of the Foundations of Nursing 2 course, in which OSCE is the summative assessment. Additionally, all the team members served as OSCE evaluators. All researchers are nurse educators familiar with the course curriculum and assessment structure, and they served as moderators and co-moderators of the focus group interviews. The researchers were aware of the students’ vulnerable positions, as their role as students might have discouraged them from withdrawing. Therefore, before each focus group interview, students were reminded that they could withdraw if they did not feel comfortable being interviewed, thereby providing them with additional opportunities to consent or withdraw from the study. None of the participants who agreed to participate in the study withdrew from the study. The participants did not receive either financial or other benefits from participating in the study. All audio files from the interviews, along with their transcripts, are stored on a password-protected personal computer in accordance with OsloMets’ data protection regulations. Only the researchers involved in the study have access to the interviews and transcripts.

### Rigor of the Study

Elo et al. (2014) emphasize that trustworthiness should be maintained throughout all stages of qualitative research. This can be achieved through the application of Lincoln and Guba’s (1985) established criteria: credibility, dependability, confirmability, and transferability. These criteria must be supported by comprehensive and transparent documentation of the entire analytical process.

In the preparation phase, key concerns include the data collection method, sampling strategy, and unit of analysis. Trustworthiness was demonstrated by clearly justifying the use of focus group interviews and purposive sampling, the latter of which was chosen based on questions about suitable informants and selection criteria (Elo et al., 2014). The diversity in participant characteristics strengthened the study by ensuring a broad range of viewpoints related to OSCE experiences. Variation in age, gender, prior healthcare experience, and examination outcomes provided insight into how students with different levels of maturity, professional exposure, and performance perceptions engaged with the OSCE. Additionally, including students from different classes and groups and conducting eight focus groups reduced the risk of capturing only group-specific norms or experiences. Together, these factors enhance the credibility and richness of the data, enabling a more comprehensive understanding of first-year nursing students’ experiences with the OSCE. Although focus group sizes varied and the number of participants was modest, the diverse characteristics of participants contributed to a range of perspectives, strengthening the transferability of the findings.

Dependability, which refers to the stability of data over time (Lincoln & Guba, 1985), was addressed by outlining inclusion criteria and participant characteristics to enhance the transferability of the findings. Credibility was further reinforced through the collaborative selection of units of analysis among the researchers. Ongoing discussions led to a consensus, thereby demonstrating transparency in the analysis process.

During the organization phase, credibility and confirmability were enhanced by ensuring that categories reflected participants’ statements rather than researchers’ interpretations. The first and last authors conducted the initial analysis. The co-authors then reviewed and discussed the categorizations until agreement was reached, thereby providing a clear rationale for category creation.

In the reporting phase, the systematic presentation of results linked data to findings, supporting credibility, transferability, and confirmability (Elo et al., 2014). Quotations are used to illustrate participants’ perspectives and enhance transparency. Furthermore, detailed descriptions of methods and analysis phases enable readers to assess the study’s trustworthiness and follow its decision-making process, thus reinforcing dependability.

### Findings

Analysis of the focus groups revealed that students’ experiences of the OSCE were likened to being caught in a storm of emotions. These experiences were grouped into three main categories: (i) Before the OSCE – The Calm Before the Storm, (ii) During the OSCE – In the Eye of the Storm, and (iii) After the OSCE – Emerging from the Storm. These categories are understood as distinct phases that reflect nursing students’ changing emotional, cognitive, and practical experiences with the OSCE as a summative assessment method. Each main category will be described in detail, along with quotes from the participants. Each quote ends with the letter “P,” indicating that the speaker is a Participant from a specific focus group (FG. . .), categorized as belonging to either the AB or CD classes.

### Before the OSCE – The Calm Before the Storm

This category reflects the students’ readiness for the OSCE exam. They often engaged in preparation and developed study strategies as a way of coping, yet this was accompanied by feelings of uncertainty and apprehension about the upcoming assessment. Despite their efforts, many continued questioning their level of readiness and self-confidence, revealing the tension between preparation, anxiety, and perceived competence.

Students’ preparation was not only a means of skill rehearsal but also a strategy to manage stress and reinforce a sense of control in the face of uncertainty. Participants described the preparation as intensive and largely self-directed, involving peer collaboration in the department’s simulation learning environment, designing checklists, and strict adherence to digital procedural guidelines, specifically the Norwegian Healthcare Online Portal. All participants acknowledged that the OSCE was a motivator in their preparation for the exam and were aware of the importance of preparing themselves to pass it. One participant said: “*Without the exam, I don’t think we would have trained this much. It [the upcoming exam] forces you to really learn*” (P, FG5, AB classes).

Despite efforts to learn and consistent preparation, many students continued to question their level of readiness and self-confidence, reflecting the complex interplay between anxiety, preparation, and perceived competence that shaped their approaches to the exam. One participant shared their frustration:

What is the correct procedure? We didn’t have a checklist, so we didn’t know what was relevant or less relevant . . . [to learn]. You know that one thing is written in the Healthcare Online Portal, but then we do different things in the simulation learning environment when we practice. What is actually the right way to do it? Where is the limit for when what I do is so wrong that I fail? (P, FG2, AB classes)

Some of them also highlighted significant structural barriers that increased their uncertainty.

For example, participants from AB-classes described uneven clinical placement experiences, ranging from limited exposure to essential procedures to being used primarily as extra staff: “*We were expected to be the ‘workforce’ in practice. I asked for time to prepare, but they said, ‘We’re understaffed’. . .*” (P, FG3, AB classes). In contrast, participants from the CD class frequently reported access to supportive clinical placements and opportunities to integrate skills in real-world settings. One participant said:

I performed NEWS [National Early Warning Score] measurements for the entire ward twice and provided fundamental care to several residents during my clinical period at the nursing home. It provided a thorough overview of what I needed. (P, FG2, CD classes)

The unit where nursing students were assigned during their clinical placement at the nursing home was crucial in providing them with chances to improve their clinical skills. This opportunity was highly valued as it could either increase or limit their chances to prepare themselves. A lack of opportunities to practice different procedures contributed to lowering their self-confidence, as one participant mentioned: “*I was on a psychiatric unit. We had limited contact with patients and performed no clinical procedures. It’s a huge disadvantage when preparing for the OSCE*” (P, FG5, AB classes).

Preparation within the department’s simulation learning environment was highly valued; however, recurring frustrations included the lack of teaching continuity and resource issues, with few opportunities to follow up with the same nurse educator. One participant said: “*We never had a consistent instructor. Each simulation session was conducted by a new instructor, so no one knew what we had learned from previous sessions. It felt unstructured and self-taught*” (P, FG5, AB classes).

Another challenge was related to opportunities for them to prepare in the department’s simulation learning environment, because other students had been there earlier and did not clean up after themselves, as one of the participants described: “*The rooms were chaotic. We spent one hour cleaning before we could even start practicing*” (P, FG2, CD classes). However, several participants mentioned that, despite chaotic rooms, they could collaboratively form peer groups, create structured checklists, and systematically distribute content to ensure full engagement with all necessary procedures. Strict reliance on the Norwegian Healthcare Online Portal also created tension between idealized, standardized procedures and clinical realities: “*I followed Healthcare Online Portal* s*lavishly because that’s what they [the evaluators] mark you on. However, in practice, some steps simply don’t work that way. It feels artificial*” (P, FG5, AB classes).

Finally, some students’ perception of poor and inconsistent information flow, especially among students from AB classes, was a significant source of stress and could potentially diminish their sense of preparedness, as one student said:

Most of the information I received just before the OSCE exam came from other students, not from the university. We trained for catheterization for days, only to find out at the last minute that it wasn’t even included [in the outlined procedures]. It’s demotivating . . . (P, FG5, AB classes)

### During the OSCE – In the Eye of the Storm

This category highlights how students navigated the complexities of the assessment process through three interconnected dimensions. They described the challenge of managing stress and maintaining composure in high-pressure situations, while simultaneously processing information and making decisions in real time. In addition to these immediate demands, students also reflected on the fairness and authenticity of the assessment, which shaped their overall perception of the experience. Together, these subcategories illustrate the multifaceted nature of students’ engagement with the assessment context.

Across all groups, the OSCE was perceived as highly stressful, with time constraints, unfamiliar settings, and silent observation from the evaluator heightening anxiety. One participant said: “*The moment I walked in and saw the evaluator just staring at me with the checklist, I almost blacked out. My hands were shaking so much I couldn’t get the needle on*” (P, FG4, AB classes). Many participants found the OSCE exam challenging to cope with. Before the assessment, several expressed the belief that a practical exam was unnecessary. Others mentioned they had never felt so nervous before. The act of standing up and performing in front of an evaluator was mentally demanding, as one participant described: “*It gives you a rush of adrenaline, which may make you perform better. It was actually a very fun exam, but it was nerve-wracking. It was the first time we had taken a practical exam!*” (P, FG1, AB classes).

Students also identified some practical barriers within the exam itself, such as unfamiliar equipment layout and unclear instructions. One participant said: “*We’d never seen that kind of glove packaging before . . . I wasted time trying to find the right size. Why not show us beforehand?*” (P, FG5, AB classes). Another participant recounted their experience: “*I thought I’d be able to start when I walked in, but they blew the whistle before I even got to my station. I lost precious time just running to the right room*” (P, FG3, AB classes). Such perceptions were less frequent in CD classes, where students often framed stress as a personal challenge to overcome rather than a symptom of systemic unfairness: “*I was nervous, sure, but it’s good practice for real-life pressure. It shows you can handle stress*” (P, FG2, CD classes).

While some students adapted as the exam progressed, as one stated: “*By the last station, I felt more in control*” (P, FG4, AB classes), many criticized inconsistencies among evaluators’ assessments: “*Some people failed due to tiny errors, while others [students] passed despite bigger mistakes. It felt arbitrary*” (P, FG3, AB classes). For most of the students, the evaluators’ mimic was interpreted in both positive and negative ways, as one of the participants explained: “*One examiner smiled and made me feel at ease; another barely looked at me. It changes everything when you feel you’re being judged harshly for no reason*” (P, FG5, AB classes).

### After the OSCE – Emerging from the Storm

This category describes students’ post-exam reflections, which revealed a complex interplay of pride, motivation, and frustration. While many valued OSCE as a powerful driver of learning and an opportunity to demonstrate competence, they also raised concerns about stress, unclear assessment criteria, and the lack of constructive feedback. The findings presented below illustrate this duality, highlighting both the perceived educational benefits of the OSCE and the students’ critique of its design, consequences, and opportunities for improvement.

Post-exam reflections combined pride in achievement with criticism of assessment design and its consequences. Many participants recognized the value of the OSCE as a strong learning motivator. One of the participants said: “*It’s an excellent way to make sure we know our stuff. I’m definitely feeling more confident now*” (P, FG2, CD classes). Another participant acknowledged that preliminary preparations contributed to learning: “*I learned more in the week leading up to the OSCE than in the months of practice that preceded it. It forced me to prepare properly*” (P, FG5, AB classes).

After the stress and uncertainty of the exam, students expressed a sense of relief and satisfaction at having completed it and demonstrated their skills. One participant said: “*I know that the purpose of the OSCE was to demonstrate to our teachers what we had learned in practice, and I felt really good about being able to show that!*” (P, FG3, AB classes).

Nevertheless, participants from AB classes in particular expressed frustration with the high stakes, unclear evaluation criteria, and lack of feedback. One participant stated: “*We don’t even know what the ‘red flags’ are. Are you failing for forgetting to label a syringe, or only for giving the wrong dose? No one tells us*” (P, FG5, AB classes). Another expressed concerns about the consequences of failing: “*Failing means retaking both the written and practical exams. It’s exhausting and unfair, especially when you failed because of something you actually did right but couldn’t prove*” (P, FG4, AB classes).

Several participants argued that the OSCE’s summative nature reduces its educational value, instead favoring formative or hybrid models, as one explained:

At [name of another university], OSCE isn’t an exam; instead, you receive feedback, and you are given the opportunity to correct mistakes. That’s how it is in real nursing: you’re not left alone, to fail. Why can’t we do the same? (P, FG5, AB classes)

Another participant suggested: “*Consider making it a pass/fail requirement or integrating feedback sessions. Right now, it feels like punishment, not learning*” (P, FG4, AB classes).

Students offered several concrete suggestions to improve the OSCE assessment. They emphasized the importance of receiving clearer and more consistent feedback during skills training and proposed involving senior students as peer mentors to strengthen preparation. Some highlighted that certain procedures, such as measuring blood pressure and pulse, require more time, suggesting that the allotted minutes should vary depending on the task, as there is a difference between washing the hands, which takes only 1 min, and measuring blood pressure and pulse. One participant described as follows:

. . . However, I believe that eight minutes is not sufficient time to complete the pulse and blood pressure measurement. You first need to say ‘hello’ to the patient and verify that it is the correct individual, and this isn’t something you can rush. It takes time, so eight minutes feel far too brief. . . (P, FG2, CD classes)

Others suggested small but significant adjustments to the conduct of the assessment and called for increased transparency in the evaluation criteria, highlighting uncertainty about which errors might result in failure: . . . “*to know what we could fail on, it wasn’t completely clear, I think. . .*” (P, FG 2, CD classes).

## Discussion

The aim of this study was to explore nursing students’ experiences with the OSCE as an assessment method for skills and competencies following the Foundations of Nursing 2 course. The findings revealed that OSCE was perceived as a strong motivator for learning and professional development, but its implementation also exposed significant disparities and challenges. Inconsistent opportunities for preparation, lack of transparency in criteria, limited psychological safety, and the high-stakes format disproportionately affected certain students. At the same time, OSCE provided a safeguard for curriculum outcomes, encouraged self-directed learning, and contributed to identity development. The discussion is organized into three main themes: “Learning – Students’ Readiness to Face the Storm,” “Feeling Psychologically Safe Within the Storm,” and “Self-development and Confidence – Surviving the Storm.” Each theme is discussed considering existing literature on the subject, emphasizing both the strengths and limitations of OSCE as a pedagogical and assessment method.

## Learning – Students’ Readiness to Face the Storm

Across the focus groups, participants emphasized that the OSCE motivated them to prepare more thoroughly to achieve learning outcomes than written exams. They described the OSCE as an assessment that demanded demonstrable competence in a live performance. This aligns with previous research, which shows that the OSCE drives student engagement and encourages in-depth preparation (Ha & Lim, 2023; Vijayalakshmi & Revathi, 2024). By linking assessment directly to the demonstration of skills, the OSCE becomes a tool of constructive alignment, where learning outcomes, teaching activities, and assessment are closely integrated (Biggs, 1996; Grande et al., 2022).

Students reported making a “bigger effort” in anticipation of the OSCE, often dedicating extra hours outside scheduled classes at the department’s simulation learning environment to rehearse procedures. Results from a study conducted among medical students revealed that voluntary practice improves the outcome of the OSCE (Tsikas et al., 2024). This motivational effect has also been confirmed in other studies, where nursing students consistently describe the OSCE as challenging yet requiring effort, but also more rewarding than traditional assessments (Huldah, 2024; Llaguno et al., 2024). However, this motivational impact had a dual nature for the participants in the current study. While many rose to the challenge, others found the OSCE overwhelming, reporting that stress and pressure undermined their concentration, knowledge recall, and perceived competence. For many participants, however, the OSCE was a moment of pride and validation of their knowledge and skills. These findings align with the existing literature, which describes assessment as both a powerful motivator and a potent stressor (Hosseini et al., 2025; Zamanzadeh et al., 2021). Furthermore, successfully demonstrating competence under scrutiny reinforced students’ sense of belonging to the nursing profession. Similar findings about students perceiving the OSCE as a rite of passage and a symbolic marker of professional identity formation are also presented in earlier studies (Alamri et al., 2022; Llaguno et al., 2024).

Clinical placements are considered essential for students’ learning and readiness for the OSCE. When given opportunities to prepare, students seek targeted guidance and rehearsal from nurse preceptors; without such preparation, they feel disadvantaged, which heightens their stress and anxiety. These findings underscore the importance of familiarizing clinical partners with academic assessment methods, as confirmed by the study of Abhinaya et al. (2024), which highlights that the OSCE can bridge academia and practice, ensuring students practice core skills regardless of placement differences. However, as the participants have expressed, inequities do occur. Results from two studies (Siddique & Shah, 2024; Zamanzadeh et al., 2021) indicate that inconsistent institutional support and varying nurse preceptors’ insufficient knowledge about students’ learning outcomes often leave students unevenly prepared for the OSCE. This raises concerns about students’ perceptions of fairness regarding the opportunities to use clinical placement as a learning context, since the OSCE is intended as a standardized exam. Strengthening partnerships with clinical partners and clearly informing nurse preceptors about the OSCE’s objectives could improve students’ readiness and also support nurse preceptors’ teaching practices.

Another outcome emphasized by participants was how the OSCE encouraged self-promoting learning in the department’s simulation learning environment. Students reported that, despite the chaotic conditions in the rooms at the department’s simulation learning environment, they successfully formed peer groups, designed checklists, and divided content to ensure comprehensive coverage of all relevant procedures. This reflective preparation fostered critical thinking, aligning with the role of OSCE as both a summative and formative assessment (Nyangeni et al., 2024). The OSCE can thus promote deep reflection and integration of knowledge, particularly when stations require clinical reasoning beyond mere procedural steps (Kassabry, 2023; Yeates et al., 2025). However, when students perceive success as primarily dependent on evaluators’ ticking off checklist items, the exam can encourage narrow, checklist-focused rehearsal (Mahmoud, 2023).

Another factor that increased participants’ stress and anxiety before the OSCE was the discrepancies in instructors’ teaching within the simulation learning environment. The absence of a consistent instructor and the lack of continuity in teaching led to students perceiving inconsistent preparation. This finding echoes calls in the literature for standardized teaching and feedback protocols in simulation learning environments, to promote fairness, consistency, and alignment across clinical settings (Chabrera et al., 2023; Dewi et al., 2024; Fuentes-Cimma et al., 2024). Faculty development is essential to help instructors strike a balance between personal expertise and curricular fidelity.

One of OSCE’s strengths is its ability to safeguard the curriculum’s outcomes. Participants appreciated this function and described OSCE as a fair method to ensure consistency across the cohort. International research also highlights the OSCE’s role in standardization and fairness (Alamri et al., 2022; Solà-Pola et al., 2020). However, standardization has proven to have some drawbacks. Participants focused primarily on the procedures most likely to appear in the Norwegian Healthcare Online Portal, as these make up the content of the OSCE. This standardization may sometimes come at the expense of broader skills such as empathy or ethical reasoning, with students strategically learning what was expected to appear in assessments at the expense of what was needed in practice (Kusurkar et al., 2023). This reflects the principle that “what is assessed becomes what is learned” (van der Vleuten et al., 2012); therefore, while securing technical competence is crucial, evaluators must balance the assessment of technical procedures during the OSCE with other assessments to fully capture the complexity of nursing practice.

The findings revealed that students emphasized the importance of clear, consistent information from the university rather than from peers, which increased confusion within the student group. Similarly, findings from a qualitative study exploring the challenges and needs of nursing students in relation to OSCE exam stress (Hosseini et al., 2025) indicate that receiving stressful and sometimes inaccurate information from classmates fosters a negative outlook and preconceptions about the exam, leading to increased stress. Therefore, universities should prioritize timely, transparent communication and structured guidance to reduce misinformation and alleviate student anxiety.

## Feeling Psychologically Safe Within the Storm

The participants identified several factors that undermined psychological safety during the OSCE: limited time, unfamiliar environments, unknown evaluators, and unfamiliar equipment. These conditions led to visible anxiety, such as shaking or freezing, which students perceived as barriers unrelated to their actual ability. This aligns with research indicating that psychological safety is essential for genuine performance in simulations and assessments (Sessions et al., 2025; Turner et al., 2023).

Time limitations can increase anxiety and stress, as students mentioned they are in a hurry and confused about the steps of procedures. Some also stated that they waste their time and become nervous during the OSCE, which is related to the “fear of the unknown,” a phenomenon similar to that observed in an earlier study (Raziani et al., 2022). The participants reported that stress peaked at the first station but declined by the last, thus indicating adaptation. While this adaptation demonstrates resilience, it also raises fairness concerns: students who struggled early may have been penalized before stress subsided. Similar observations are mentioned in the literature, which describes how stress responses fluctuate during the OSCE (Hansen et al., 2023; Mauriz et al., 2021). However, several participants agreed that training and repeating procedures helped reduce stress and anxiety and improved performance. They described that stress could be channeled productively when combined with preparation, which aligns with the idea that moderate stress enhances engagement (Dieckmann et al., 2009; Hansen et al., 2023). In this sense, the OSCE not only assessed competence but also trained students in stress management, a skill that is transferable to clinical practice.

As the findings revealed, not all students responded positively to the OSCE as a form of assessment. Some questioned its purpose, found the criteria unclear, or perceived it as unfair. The stress arose as much from the novelty of the OSCE as from its content. These concerns have been reported in early studies (Hosseini et al., 2025; Zamanzadeh et al., 2021). At the same time, such perceptions may also indicate incomplete integration of learning outcomes. When students cannot link the assessment to the learning outcomes, they may feel disconnected (Hardie et al., 2022). However, results from several studies (Braier-Lorimer & Warren-Miell, 2022; Fuladvandi et al., 2024; Gilani et al., 2022; Lim et al., 2023) indicate that mock OSCE helps students reduce unnecessary anxiety by familiarizing them with the exam format, timing, and expectations, enhances students’ preparedness, confidence, and performance; therefore, it is advisable to introduce mock OSCE into education as early as possible. Although resource-intensive, a mock OSCE can help ensure that the OSCE assesses competence rather than merely coping with its unfamiliarity and doubting its purpose (Fuladvandi et al., 2024).

A reflective question is whether “stress” sometimes acts as a protective factor and as an explanation for poor performance. Without knowing exam results, it is uncertain whether stressed students were underprepared. Studies on challenges in OSCE (Siddique & Shah, 2024; Zamanzadeh et al., 2021) suggest that students often interpret structural issues as personal stress. However, the findings from the current study demonstrate that excessive stress impaired performance. Similar results were also revealed in other studies (Adibone Emebigwine et al., 2023; Yeh & Yang, 2024). The participants, therefore, felt that the OSCE assessed both competence and stress tolerance, raising questions about its fairness. Educators must therefore distinguish between stress that can be alleviated through customized exam designs and stress that indicates inadequate preparation.

Students’ ability to perform under stress, process information quickly, and make decisions with incomplete data are professional competencies just as much as exam skills. Struggles in OSCE may indicate broader learning needs, while passing signals readiness for clinical practice. The OSCE, therefore, functions both as an exam and as a rehearsal for professional behavior (Cömert et al., 2016; Mauriz et al., 2021).

As the findings revealed, evaluators’ behavior during student assessments also contributed to increased stress among students. The assessment was perceived as unfair, as some students made mistakes and still passed, while others failed. Similar to the findings from the study conducted by Raziani et al. (2022), which indicate that the evaluator’s behavior can impact students’ stress levels during the OSCE, the participants in the current study reported that cold or distant evaluators heightened anxiety, while professional yet welcoming evaluators reduced it. Training evaluators in communication, neutrality, and professionalism is therefore essential (Guerrero et al., 2024). Standardized training among evaluators would ensure consistency across stations, fairness in assessment, and minimize variability in students’ stress responses.

## Self-Development and Confidence – Surviving the Storm

The participants regarded the OSCE as a significant milestone that validated their learning progress and inspired them to pursue further learning. Preparing for the OSCE aimed not only to enhance technical skills but also soft skills such as communication, teamwork, time management, and perseverance (Ahmad et al., 2025). Despite feeling stressed and worried about their level of preparation, passing the exam boosted students’ confidence and self-efficacy, and they experienced a sense of relief. Similar findings are reported in other studies (Thabet et al., 2021; Vijayalakshmi & Revathi, 2024), indicating that the OSCE improves students’ beliefs in their professional readiness.

Beyond specific skills, students emphasized that the OSCE taught them how to learn. Preparing for the OSCE involved planning, repetition, and self-assessment, fostering meta-cognitive strategies that are applicable to future learning (Nyangeni et al., 2024). This aligns with literature describing the OSCE as a method that teaches “learning to learn” and supports reflective practice (Régent et al., 2023; Rudolph et al., 2008).

The findings revealed that some participants expressed frustrations with, among other things, the evaluation criteria. In this program, students were not given explicit lists of OSCE criteria for passing. Instead, they were expected to infer them from coursework and clinical practice. This was intended to foster critical thinking. Some students indeed reported deeper engagement, while others felt insecure. The tension between explicit and implicit criteria, with explicit criteria increasing transparency and implicit criteria promoting judgment and critical reasoning, is well-documented (Khong & Tanner, 2024). Explicit assessment criteria (e.g., rubrics, tables or detailed checklists) are frequently advocated to increase transparency, clarify expectations, and reduce student anxiety (Jönsson & Prins, 2019). However, overly prescriptive criteria may limit deeper reasoning, and it is argued that less explicit, more tacit, or holistic criteria allow students to apply judgment and foster critical reasoning (Bearman & Ajjawi, 2021). In the context of nursing, the absence of clearly communicated criteria has been associated with perceptions of unfairness in clinical assessment settings (Alkhelaiwi et al., 2024). However, what is important is that students’ critiques demonstrate their ability to reflect critically. By questioning clarity, fairness, and stress, students demonstrated evaluative reasoning, a crucial professional skill (Zamanzadeh et al., 2021). Similar findings are reported by Alizadeh et al. (2024), who found that nursing students’ frustrations around the OSCE often coexisted with recognition of gaps in their practice and the need to meet professional standards, indicating a growing awareness of professional responsibilities. Therefore, what appears to be resistance may actually indicate growth.

Although the OSCE was primarily a summative exam, students still strongly desired feedback afterwards. For the students who passed, feedback was generally perceived as affirming their performance and helping to consolidate learning for future practice. For students who did not pass and needed debriefing, evaluators provided feedback that, although sometimes perceived as discouraging or unclear, served as an important guide for future improvement. In their study, Jay et al. (2023) found that emotions such as anger, fear, anxiety, and sadness were commonly experienced following examination failure among medical students. The medical students reported a disconnect between what they believed feedback should provide and the actual benefits it offered after summative assessment. Results from other studies show that feedback is necessary because it provides closure, highlights strengths, guides future learning, and improves self-efficacy, emotional intelligence, and learning strategies (Daniels et al., 2021; Ngim et al., 2021; Thabet et al., 2021). However, giving feedback to large cohorts (almost 550 students annually) is logistically challenging. Solutions such as digital platforms, standardized rubrics, or small-group debriefings have been proposed (Ngim et al., 2021).

As evaluators, nurse educators should value student feedback but also interpret it critically. Students’ preparedness depends not only on exam design but also on student engagement in learning, a statement emphasizing the shared responsibility between educators and learners (van der Vleuten et al., 2012). While student critiques should inform improvement, they also highlight the necessity of student engagement. Ultimately, meaningful assessment practices require a balance between educator accountability and learner responsibility, ensuring that feedback fosters growth rather than reinforcing dissatisfaction.

## Strengths and Limitations of the Study

The study has several notable strengths. First, its qualitative design enables an in-depth exploration of students’ experiences, emotions, and perceptions, beyond what can be captured through quantitative measures. By using focus groups, the researchers created opportunities for participants to discuss and reflect collectively, fostering rich and interactive dialogues that often lead to deeper insights. The inclusion of eight focus groups provides a wide range of data, increasing the likelihood of capturing diverse perspectives across the student cohort. Furthermore, involving multiple researchers with experience in clinical nursing, nursing education, curriculum development, and extensive experience in qualitative methodologies enhances the study’s credibility. Their combined skills help ensure data are collected sensitively, participants’ views are thoroughly explored, and findings are rigorously analyzed through qualitative content analysis. The researchers’ triangulation also improves trustworthiness, reducing the risk that individual bias may unnecessarily influence interpretation.

Nevertheless, the study has several limitations. Focus groups, though useful for fostering discussion, can be influenced by group dynamics, with quieter students potentially overshadowed by more outspoken peers. This could limit the extent to which all perspectives are equally represented. We recognize that relying solely on focus groups may pose a risk of opinion convergence, especially when there are dominant voices. To address this, the moderator actively promoted participation from all students in the group, facilitated turn-taking, and asked follow-up questions to draw out each individual’s perspectives. While individual interviews were considered, given the exploratory nature of the study and the pedagogical focus of the topic, focus groups were deemed the most suitable method for encouraging shared reflection and capturing the interactional dynamics concerning OSCE experience. Conducting focus groups was also a time-saving method for data collection, as it allowed us to gather a wide range of student perspectives efficiently within a limited timeframe.

A potential limitation is that member checking was not conducted. Participants from the AB class started a new course immediately after the OSCE, while those from the CD class began their summer holidays only 1 week later. Consequently, it was not feasible to request their feedback on the interview transcripts or on the data analysis at a later stage. However, to enhance credibility despite this limitation, the moderators actively encouraged participants to elaborate on their responses, asked clarifying questions, and summarized key points during the focus groups. This allowed participants to confirm or refine the interpretations in real time, thereby providing an alternative form of validation.

Another potential limitation is that the study relies on first-year students’ immediate reflections after the OSCE, which may have been influenced by stress or relief, potentially biasing the data toward more emotional reactions. Additionally, the generalizability and transferability of the findings may be limited, as OSCE structures and student experiences can vary significantly across institutions. Therefore, the reflections from first-year students at OsloMet may not fully represent the perspectives of students in other educational settings.

Finally, while using multiple researchers is a strength, it also introduces the potential for variability in facilitation styles and interpretive frameworks, which, if not carefully managed, could impact consistency across the focus groups and in the analysis. To address this, the researchers discussed the analyses and the findings until a consensus was reached, ensuring a shared understanding of the data. In practice, both the initial analysis and interpretation of the findings were primarily undertaken by the first and last authors, which helped maintain consistency while still benefiting from the wider insights of the co-authors.

Overall, the study provides a valuable and credible account of students’ experiences during their first OSCE. Furthermore, the study contributes new insights to the field of nursing education by highlighting how the OSCE not only functions as an assessment tool but also shapes students’ professional identity, self-reflection, and perception of clinical readiness. While earlier studies have primarily confirmed OSCE’s role in generating stress and providing a standardized evaluation format, our findings emphasize its dual role as both a learning arena and a transformative experience. This suggests that OSCE may contribute more directly to bridging the gap between theoretical knowledge and the complexities of real-life clinical practice than previously documented.

## Implications for Nursing Education

To enhance the effectiveness of clinical education, stronger alignment between academia and clinical placements is essential. Clinical mentors should be fully informed about the curriculum and the outcomes of the OSCE exam so they can better support students in their preparation. In addition, providing formative opportunities such as mock OSCEs or low-stakes simulations may help familiarize students with the examination format and reduce performance-related anxiety.

Consistency is another critical factor. Standardizing both teaching and assessment requires targeted training for educators and evaluators to ensure uniform expectations across teaching stations and evaluation procedures. Peer learning can also be leveraged, involving senior students as mentors, which not only extends opportunities for practice but also fosters a supportive and collaborative learning community.

Finally, effective feedback mechanisms must be prioritized. Even in large student cohorts, scalable feedback strategies are essential for maintaining the formative value of the OSCE and supporting students’ ongoing development. By addressing these areas, the OSCE can evolve from being merely a high-stakes test into a balanced assessment that both evaluates and encourages student competence.

## Conclusion

This study demonstrates that OSCEs are a valuable pedagogical tool for use in summative assessments of nursing students’ learning. It motivates the learning of nursing procedures through preparation, promotes critical thinking, ensures curricular outcomes, and contributes to the development of a professional identity. However, it also risks fostering inequality, surface learning, and excessive stress and anxiety among students. Addressing these challenges requires better alignment between academia and clinical placements, standardized teaching protocols, peer-learning opportunities, mock OSCEs, feedback mechanisms, and evaluator training.

Overall, the OSCE was experienced as a “storm” of feelings, both empowering and frustrating. For many, it validated growth, boosted confidence, and encouraged further learning, ultimately fostering a sense of confidence. For others, it raised questions about the clarity and fairness of the assessment, thus leading to frustration. Nonetheless, even the critique demonstrated critical reflection and the development of professional identity. The OSCE thus functions as more than just an exam: it is a reflection of students’ learning motivation, resilience, and readiness for the nursing profession. Ultimately, the OSCE outcomes reflect not only what students know but also who they are becoming as nurses. Their ability to manage stress, engage critically, and perform under pressure signals readiness not just for the exam, but also for the nursing profession.
